# A Rapid Antigen Detection Test to Diagnose SARS-CoV-2 Infection Using Exhaled Breath Condensate by A Modified Inflammacheck^®^ Device

**DOI:** 10.3390/s21175710

**Published:** 2021-08-25

**Authors:** Mauro Maniscalco, Pasquale Ambrosino, Anna Ciullo, Salvatore Fuschillo, Valerio Valente, Carlo Gaudiosi, Debora Paris, Raffaele Cobuccio, Francesco Stefanelli, Andrea Motta

**Affiliations:** 1 Istituti Clinici Scientifici Maugeri IRCCS, 27100 Pavia, Italy; pasquale.ambrosino@icsmaugeri.it (P.A.); anna.ciullo@icsmaugeri.it (A.C.); salvatore.fuschillo@icsmaugeri.it (S.F.); Valerio.valente@icsmaugeri.it (V.V.); 2 Department of Respiratory Medicine, Boscotrecase Covid Hospital, 80042 Boscotrecase, Italy; c.gaudiosi@aslnapoli3sud.it (C.G.); pobosc.pneumo@aslnapoli3sud.it (R.C.); f.stefanelli@aslnapoli3sud.it (F.S.); 3 Institute of Biomolecular Chemistry, National Research Council, ICB-CNR, 80078 Pozzuoli, Italy; debora.paris@icb.cnr.it (D.P.); andrea.motta@icb.cnr.it (A.M.)

**Keywords:** COVID-19, SARS-CoV-2, exercise, disability, rehabilitation, outcome

## Abstract

Background: The standard test that identifies the severe acute respiratory syndrome coronavirus-2 (SARS-CoV-2) is based on reverse transcriptase-polymerase chain reaction (RT-PCR) from nasopharyngeal (NP) swab specimens. We compared the accuracy of a rapid antigen detection test using exhaled breath condensate by a modified Inflammacheck^®^ device with the standard RT-PCR to diagnose SARS-CoV-2 infection. Methods: We performed a manufacturer-independent, cross-sectional, diagnostic accuracy study involving two Italian hospitals. Sensitivity, specificity, positive (PLR) and negative likelihood ratio (NLR), positive (PPV) and negative predictive value (NPV) and diagnostic accuracy with 95% confidence intervals (95% CI) of Inflammacheck^®^ were calculated using the RT-PCR results as the standard. Further RT-PCR tests were conducted on NP specimens from test positive subjects to obtain the Ct (cycle threshold) values as indicative evidence of the viral load. Results: A total of 105 individuals (41 females, 39.0%; 64 males, 61.0%; mean age: 58.4 years) were included in the final analysis, with the RT-PCR being positive in 13 (12.4%) and negative in 92 (87.6%). The agreement between the two methods was 98.1%, with a Cohen’s κ score of 0.91 (95% CI: 0.79–1.00). The overall sensitivity and specificity of the Inflammacheck^®^ were 92.3% (95% CI: 64.0%–99.8%) and 98.9% (95% CI: 94.1%–100%), respectively, with a PLR of 84.9 (95% CI: 12.0–600.3) and a NLR of 0.08 (95% CI: 0.01–0.51). Considering a 12.4% disease prevalence in the study cohort, the PPV was 92.3% (95% CI: 62.9%–98.8%) and the NPV was 98.9% (95% CI: 93.3%–99.8%), with an overall accuracy of 98.1% (95% CI: 93.3%–99.8%). The Fagan’s nomogram substantially confirmed the clinical applicability of the test in a realistic scenario with a pre-test probability set at 4%. Ct values obtained for the positive test subjects by means of the RT-PCR were normally distributed between 26 and 38 cycles, corresponding to viral loads from light (38 cycles) to high (26 cycles). The single false negative record had a Ct value of 33, which was close to the mean of the cohort (32.5 cycles). Conclusions: The modified Inflammacheck^®^ device may be a rapid, non-demanding and cost-effective method for SARS-CoV-2 detection. This device may be used for routine practice in different healthcare settings (community, hospital, rehabilitation).

## 1. Introduction

The new severe acute respiratory syndrome coronavirus-2 (SARS-CoV-2) first appeared in December 2019, and it subsequently spread rapidly worldwide causing a global pandemic [1]. When symptoms are experienced, infected patients develop the coronavirus disease 2019 (COVID-19) [2]. To effectively reduce the spread of the infection in addition to containment measures, sensitive, specific, rapid, inexpensive and easy-to-use tests able to rule out or to confirm SARS-CoV-2 infection, possibly even in the asymptomatic phase of the infection, are welcomed.

Before SARS-CoV-2 infection manifests itself, there is usually an incubation period lasting up to 14 days, with a median of approximately 5 days from exposure to symptom onset [3]. The early identification of suspected cases represents a key strategy to control the spread and the mortality rate of the infection. Therefore, rapid, effective and costless diagnostic tests should be implemented with large-scale automated diagnostic equipment.

Currently, the reference standard test accepted for identification of SARS-CoV-2 positive subjects is based on molecular assays detecting viral RNA by reverse transcriptase-polymerase chain reaction (RT-PCR) from nasopharyngeal (NP) swabs [4]. Most diagnostic molecular tests have been approved by the United States Food and Drug Administration under emergency use authorization (EUA) and are Conformité Européenne marked [5]. These assays target various genes that include spike (S) surface glycoprotein, small envelope (E) protein, nucleocapsid (N) protein and RNA-dependent RNA polymerase (ORF1ab) [6]. To enhance test sensitivity and specificity, it has been suggested to target at least two genes [7]. However, a NP swab negative result does not necessarily rule out infection. In subjects with a negative NP swab but with a high clinical suspicion for COVID-19, to resolve the diagnostic doubt, it may be required to sample from the lower respiratory tract [8]. Molecular tests usually require qualified personnel and specialised laboratory equipment and are burdened by the potential exposure to the virus of healthcare professionals who take and process the biological samples. Definitely, the cumbersome procedures necessary for the diagnosis of SARS-CoV-2 infection represent in themselves an additional burden of the pandemic. 

SARS-CoV-2 may spread via small droplets in breath and aerosols [9] and there is evidence supporting the presence of the virus in exhaled breath condensate (EBC). Breath droplets with aerodynamic diameter lower than 5 μm are collected by EBC rendering it a suitable matrix for SARS-CoV-2 detection [10]. The angiotensin converting enzyme-2 (ACE-2) protein has been recognized as an important binding protein for SARS-CoV-2, allowing the virus to enter cells [11]. Therefore, a test detecting the virus in EBC by exploiting its ability to bind this protein may be a useful tool for a rapid diagnosis. The modified Inflammacheck^®^ device is a novel, hand-held, fully automated in vitro diagnostic medical device for non-invasive detection of SARS-CoV-2 in exhaled breath at point of care. It integrates the safe collection of EBC with an automated platform for SARS-CoV-2 detection through an embedded electrochemical sensor. The sensor surface is coated with a macromolecule to which the virus binds, producing a measurable signal indicating whether SARS-CoV-2 is present or not. The test assay renders a result after less than 6 min. EBC is collected by the Inflammacheck^®^ device through normal breathing, making this investigation non-invasive, rapid, reliable and easy to perform. The aim of our study was to compare the diagnostic sensitivity and specificity of the Inflammacheck^®^ device for SARS-CoV-2 detection with the standard RT-PCR of NP swab specimens.

## 2. Materials and Methods

We carried out a cross-sectional multicentre study across two independent hospitals of Campania Region in Italy, namely Pulmonary Rehabilitation Unit of Istituti Clinici Scientifici Maugeri Spa SB, IRCCS of Telese Terme, Benevento, and Boscotrecase Hospital, Boscotrecase, Naples. We enrolled four groups of patients at different stages of the diagnostic/screening process for potential SARS-CoV-2 infection: group 1, subjects with clinically suspected COVID-19; group 2, convalescent COVID-19 patients undergoing pulmonary rehabilitation for persistent pulmonary and/or functional limitations within four weeks from acute hospital discharge; group 3, asymptomatic subjects with a high probability of SARS-CoV-2 infection; group 4, asymptomatic subjects with a low probability of SARS-CoV-2 infection. The level of clinical suspect was established by physicians, based on the presence/absence of typical COVID-19 symptoms (i.e., fever, dry cough, shortness of breath, lethargy, myalgia, arthralgia) and of compatible radiological findings on chest X-ray or chest computed tomography (CT) scans. The major inclusion criteria were age ≥ 18 years and informed consent signature. Exclusion criteria were: invasive or non-invasive ventilation, high-flow oxygen, symptomatic hypoxia or oxygen saturation ≤ 92% (≤88% in chronic obstructive pulmonary disease) despite oxygen supplementation at 4L/min via nasal cannula, suspicion of alcohol or drug abuse or any other condition associated with poor compliance in the investigator’s opinion. The study was approved by the Institutional Review Board of Istituto Nazionale Tumori, Fondazione Pascale, Naples, Italy with reference number 2/21. All patients provided written informed consent.

### 2.1. RT-PCR Swabs

RT-PCR was performed as the reference standard on the same day of the index test using the NeuMoDx™ 300800 (Qiagen, Hilden, Germany), with results inferred according to the manufacturer’s specifications. The NeuMoDx™ SARS-CoV-2 assay is an automated sample-to-answer assay that received emergency use authorization from the FDA. It is a multiplex assay targeting two regions of the viral genome, namely Nsp2 and the N genes. The NeuMoDx™ System automatically performs all the steps required to extract the target nucleic acid and prepare the isolated RNA for RT-PCR and, if present, amplifies and detects target sequences of the Nsp2 N genes. The NeuMoDx™ System uses a combination of heat, lytic enzyme and extraction reagents to automatically perform lysis, RNA extraction and removal of inhibitors using the separately available NeuMoDx™ reagents. The released nucleic acids are captured by paramagnetic particles. The particles, with bound nucleic acid, are loaded into the “NeuMoDx™ Cartridge where the unbound elements are washed away with NeuMoDx™ Wash Reagent”. The bound RNA is then eluted using NeuMoDx™ Release Reagent. The NeuMoDx™ System uses the eluted RNA to rehydrate proprietary NeuDry™ amplification RT-PCR mix containing all the elements necessary for amplification of both SARS-CoV-2 and sample process control (SPC2) targets. The amplified targets are detected in real time using hydrolysis probe chemistry (commonly referred to as TaqMan^™^ chemistry) using fluorogenic oligonucleotide probe molecules specific to the amplicons of their respective targets. TaqMan^™^ probes are designed such that they anneal within a DNA region amplified by a specific set of primers. The Nsp2 region is detected by means a TaqMan^™^ probe labeled with a FAM fluorophore (470/510 nm) while the N gene is detected by using a TaqMan^™^ probe labeled with a HEX fluorophore (530/555 nm). A TaqMan^™^ probe labeled with a Far-Red fluorophore (680/715 nm) is used for SPC2 detection. When amplification is complete, the NeuMoDx™ System software analyzes the data and expresses the result as positive if one or both target genes have been detected, and as negative if both target genes are not amplified while SPC2 is, as undetermined in the case of an instrumental error or, finally, as unresolved in the case of the instrument’s inability to express a valid result. Quantification of the viral RNA load was achieved by employing the comparative threshold cycle (Ct) method. Ct is defined as the PCR cycle at which the accumulated amount of PCR product achieves an arbitrary threshold. This method allows for precise determination of the viral RNA load in a sample, relative to that of a positive control with known viral RNA load by comparing the Ct of the sample and the positive control included in the PCR assay kit.

### 2.2. Inflammacheck^®^

**Figure 1:**
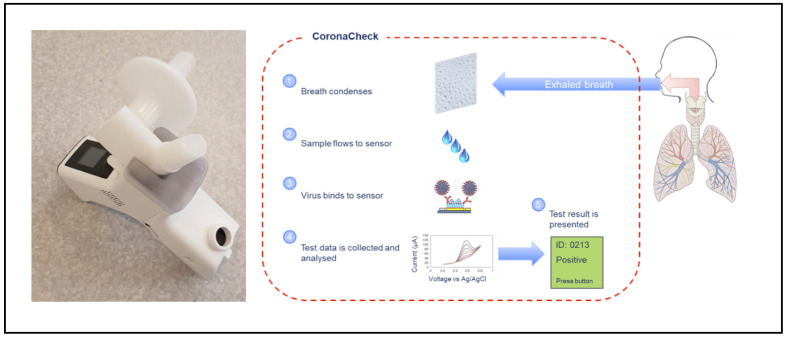
The Inflammacheck^®^ device and its operating mechanism.

When testing with the Inflammacheck^®^, the subject breathes into a disposable breath collection unit, which is mounted in the Inflammacheck^®^ device (Exhalation technology LTD, Cambridge, UK). The breath collection unit is sealed off towards the rest of the Inflammacheck^®^ system to mitigate the risk of cross-infection. It is composed of a sensor cartridge with a built-in single-use sensor, a valve house directing the airflow through the system and a bacterial viral filter. The filter serves to filter ambient air before it is inhaled, as well as to filter exhaled breath before it leaves the breath collection unit. To perform a test, the Inflammacheck^®^ is switched on, and the breath collection unit is inserted. It has an embedded RFID tag containing information on the sensor and sensor cartridge, including manufacturing batch and dates, which is read by the device and stored along with the test data establishing traceability in the system. The device performs a self-check to ensure the system is fully functional and capable of performing a test, and then initiates cooling by means of a Peltier element to bring down the temperature at the condensation area to below 6 °C. When this temperature has been reached, the device emits a beep and tells the subject to start breathing through the mouthpiece of the breath collection unit. When exhaling, the subject breathes onto a condensation area, which is part of the sensor cartridge, to form a test sample of EBC. During sample collection, EBC builds up in the condensation area until enough has been collected (20–30 µL, which typically forms in 45–90 s). Then it automatically flows into a sensor capillary. The device monitors the fill of the sensor capillary to ensure an adequate amount of sample is present to perform a test. Upon registered fill, the device emits a beep and instructs the subject to stop collecting the sample and place the device on a table. The device automatically proceeds into the analytical assay. The device performs a first test scan to establish a baseline pre-curve, and then proceeds into a 5 min incubation period where the virus, if present in the EBC, binds to the macromolecule coated sensor surface. After incubation, a second test scan is performed to build a post-curve. The presence of virus is determined by comparing pre- and post-curves. Less than 6 min after fill detection, the test result is presented back to the operator/test subject via the built-in display on the device (Figure 1). 

After use, the test consumable is disposed of in a safe bin. The sample collected is contained inside the disposable test consumable, thereby mitigating the risk of contamination between samples as well as the risk of a test subject being exposed to residuals of samples from previous test subjects. The reader device itself is wiped off between tests with 70% alcohol to further mitigate the risk of cross-infection between test subjects.

### 2.3. Statistics

Sensitivity, specificity, positive (PLR) and negative likelihood ratio (NLR), positive (PPV) and negative predictive value (NPV) and diagnostic accuracy with 95% confidence intervals (95% CI) of Inflammacheck^®^ were calculated using the RT-PCR results as standard. The level of agreement between the tests was evaluated using the Cohen’s κ score [12]. Diagnostic performance results were stratified by age and gender. Statistical analysis was performed with R Statistical software (R Core Team 2021). Fagan’s nomogram was used to estimate post-test probability from likelihood ratios and evaluate the clinical applicability of the device.

## 3. Results

Of 119 patients screened for eligibility, six (5.0%) were ineligible for protocol adherence issues. Four (3.4%) refused to participate in the study. A total of four (3.7%) out of the 109 eligible patients were not considered because measurement failed due to technical problems. Thus, 66 participants from Istituti Clninci Scientifici Maugeri and 39 from Boscotrecase Covid Hospital entered the study. Overall, a total of 105 individuals (41 females, 39.0%; 64 males, 61.0%; mean age 58.4 years) were included in the final analysis, of which 21 (20.0%) were subjects with clinically suspected COVID-19 (group 1), 20 (19.0%) convalescent COVID-19 patients (group 2), 14 (13.3%) were asymptomatic participants with a high clinical probability of SARS-CoV-2 infection (group 3) and 50 (47.6%) were asymptomatic participants with a low clinical probability of SARS-CoV-2 infection (group 4) (Table 1).

Among the 105 subjects, RT-PCR was positive in 13 (12.4%) and negative in 92 (87.6%). One 57-year-old male participant tested positive with RT-PCR but negative with the Inflammacheck^®^ device. A further discrepancy was a 31-year-old woman who tested a false positive on Inflammacheck^®^. Thus, similarly to RT-PCR, the test performed with the Inflammacheck^®^ device was positive in 13 (12.4%) cases and negative in 92 (87.6%) cases in our study sample (Table 2).

We also extracted and recorded Ct values from the NeuMoDx™ System (Table 3) obtained for the positive test subjects to assess viral load. The Ct values were distributed normally between 26 and 38 cycles, with a mean of 32.5 cycles. This was assessed to correspond to viral loads ranging from light (38 cycles) to high (26 cycles). The single false negative record had a Ct value of 33 that was close to the mean of the cohort. 

**Figure 2:**
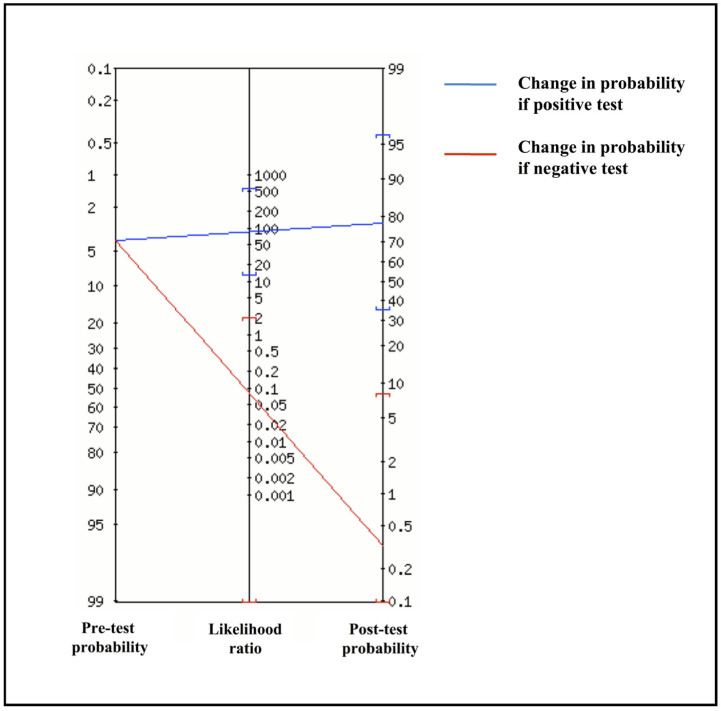
Fagan’s nomogram from positive (PLR) and negative likelihood ratio (NLR).

Based on these data, the agreement between the two methods was 98.1%, with a Cohen’s κ score of 0.91 (95% CI: 0.79–1.00). The overall sensitivity and specificity of the Inflammacheck^®^ were 92.3% (95% CI: 64.0%–99.8%) and 98.9% (95% CI: 94.1%–100%), respectively, with a PLR of 84.9 (95% CI: 12.0–600.3) and an NLR of 0.08 (95% CI: 0.01–0.51). Considering a 12.4% disease prevalence in the study cohort, the PPV was 92.3% (95% CI: 62.9%–98.8%) and the NPV was 98.9% (95% CI: 93.3%–99.8%), with a diagnostic accuracy of 98.1% (95% CI: 93.3%–99.8%). Since PPV, NPV and diagnostic accuracy can vary depending on prevalence data, these three indicators were also estimated in a realistic scenario reflecting the SARS-CoV-2 positivity rate in Italy at the time of the examination. Thus, with an estimated 4% disease prevalence, the PPV and the NPV were 78.0% (95% CI: 33.4%–96.2%) and 99.6% (95% CI: 97.9%–99.9%), respectively, with an overall accuracy of 98.6% (95% CI: 94.1%–99.9%). Accordingly, based on likelihood ratios and the pre-test probability set at 4%, the Fagan’s nomogram confirmed that the post-test probability increased to 78% if the patient tested positive and decreased to 0.4% in case of a negative result with the Inflammacheck^®^ device (Figure 2).

Table 4 shows results stratified by age and gender. Given the relatively low number of participants, no definite conclusion could be drawn in this regard.

## 4. Discussion

Results of our preliminary study suggest that the modified Inflammacheck^®^ device, as compared to NeuMoDx™ System RT-PCR NP swab, identifies SARS-CoV-2 in EBC excellently, with sensitivity and specificity of 92.3% and 98.9%, respectively. Moreover, the analysis of the likelihood ratios and the Fagan’s nomogram suggest that the test could be considered suitable for routine practice. 

Currently, RT-PCR assay represents the gold standard for diagnosis of SARS-CoV-2 infection and can be performed on nasopharyngeal and oropharyngeal swabs in symptomatic and asymptomatic people. However, RT-PCR analysis is time consuming and requires dedicated laboratory equipment and personnel specifically trained. Up to 54% of COVID-19 patients may test negative on the initial NP swab samples RT-PCR [13]. Such a high rate of false negatives results from several causes among which the most frequent are methodological errors with inadequate sample collection, errors in sampling times or errors in the handling and shipment of the sample [14]. In addition, it has been reported that certain deletions/mutations in the SARS-CoV-2 genome can affect PCR performance [15]. It is now clear that infected subjects can transmit the virus during the asymptomatic phase of infection, the viral load peak occurring one or two days before onset of symptoms [16]. After the appearance of COVID-19 symptoms, the percentage of success of the NP swab detection progressively decreases over time. In a study by Wölfel et al, all swabs taken between day 1 and day 5 tested positive, while after five days of symptoms the detection rate dropped up to 40% [17]. Current evidence supports the feasibility of detecting SARS-CoV-2 RNA in EBC by RT-PCR testing for the diagnosis of COVID-19 within two days of disease onset in spontaneously breathing subjects [9]. In a study by Ryan et al [18], it was reported that after a median number of seven symptomatic days (range 2–20), depending on the type and number of the viral genome regions targeted, the success detection rate of SARS-CoV-2 by RT-PCR in EBC was between 66.6% and 93.3% of NS-negative patients with a clinical diagnosis of COVID-19. Negative results of two consecutive molecular assays from respiratory specimens more than 24 h apart have been the suggested key strategy for discontinuation of transmission [4]. However, SARS-CoV-2 RNA can be detectable for weeks after the onset of symptoms and this does not necessarily indicate the presence of infectious virus [19,20]. Because asymptomatic infected subjects are frequent and, as compared to symptomatic patients, carry out a similar viral load in respiratory specimens [21], they could represent the main culprits of the epidemiologic worsening of the pandemic [22]. Consequently, the availability of a rapid and sensitive test for the detection of SARS-CoV-2 would overcome the diagnostic uncertainty and allow for the rapid implementation of infectious prevention measures in different healthcare settings (i.e., community, hospital, rehabilitation) [23,24,25]. The results we present here indicate that Inflammacheck^®^ permits a non-invasive detection of SARS-CoV-2 with high sensitivity and specificity in less than 6 min. In addition, EBC collection does not require manipulation of the sample as it is collected by the device itself through normal breathing, making this investigation non-invasive, rapid, reliable and easy to perform. Collecting EBC specimens has several advantages over NP swabs, among which are a lower risk of viral spread and/or environmental contamination and, above all, minimal training is required and the method can be carried out by anyone. In addition, EBC sampling can repeatedly be performed as many times as needed in follow-up investigations. Thanks to the social measures implemented and the availability of effective vaccines, the pandemic epidemiological curve has progressively improved. However, the abolition of social restrictions, together with the resumption of international travel as well as the appearance of new viral variants make the rapidity and specificity of diagnostic tests extremely important to identify and isolate in a very short time all infected subjects in the asymptomatic phase.

A major limitation of our study should be addressed. In detail, a diagnostic accuracy study should ideally enroll a random or consecutive sample of eligible patients with the suspected condition to avoid the case-control design and, therefore, a potential bias. Thus, studies enrolling patients with the target condition and a control group without it may overestimate diagnostic accuracy. Moreover, our choice of enrolling four patient groups at different stages of the diagnostic/screening process for potential SARS-CoV-2 infection accounts for the different pre-test probability and, therefore, for the impossibility of performing sensitivity or subgroup analyses other than those related to major demographic variables. Overall, caution is required in interpreting our results, which should be considered preliminary.

## 5. Conclusions

Our preliminary results demonstrate that Inflammacheck^®^ enables the qualitative detection of SARS-CoV-2 in EBC samples in a reliable manner, with excellent overall, positive and negative percentage agreements and with a high κ value. Owing to its simplicity and high sensitivity, Inflammacheck^®^ is particularly suited in the triage process and for a rapid screening of a large population, which would be useful at airports, on college campuses, drive-through screening clinics, shopping centres, train stations, point of care facilities, etc. However, the results we presented should be validated in a larger cohort study, and the performance of the device in differentially diagnosing SARS-CoV-2 from similar respiratory viruses should also be checked.

## Figures and Tables

**Table 1:** Study cohort included in the validation study of the Inflammacheck^®^ device.

Total Number of Valid RT-PCR Tests	105
Positive RT-PCR, n (%)	13 (12.4)
Age (years)	58.4 ± 14.4
Female gender (%)	41 (39.0)
Clinically suspected COVID-19, n (%)	21 (20.0)
Convalescent COVID-19, n (%)	20 (19.0)
Asymptomatic with high clinical probability, n (%)	14 (13.3)
Asymptomatic with low clinical probability, n (%)	50 (47.6)

RT-PCR: Reverse transcriptase-polymerase chain reaction; COVID-19: coronavirus disease 2019; n: number.

**Table 2:** Summary of the results of the Inflammacheck^®^ device compared to standard RT-PCR from nasopharyngeal swab specimens.

RT-PCR	Inflammacheck^®^ Device		
	Positive	Negative	Total
Positive	12	1	13
Negative	1	91	92
Total	13	92	105

**Table 3:** Summary of the results of the Ct values of each positive subject included in the study obtained with NeuMoDx™ System. Age and gender included for statistical reasons.

Positive Subject	Age	Gender	Study Group	Ct Value
1	76	Female	1	33
2	70	Male	1	28
3	49	Male	1	33
4	41	Male	1	32
5	78	Male	1	31
6	54	Male	1	38
7	65	Male	1	26
8	68	Male	3	28
9	59	Female	1	34
10	74	Male	3	38
11	44	Male	1	37
12	57	Male	1	33
13	36	Male	1	31

**Table 4:** Estimation of clinical performance of the Inflammacheck^®^ device compared to standard reverse transcriptase-polymerase chain reaction (RT-PCR) from nasopharyngeal swab specimens.

	Value	95% CI
Total cohort (*n* = 105)		
Sensitivity	92.3%	64.0% to 99.8%
Specificity	98.9%	94.1% to 100%
Positive Likelihood Ratio	84.9	12.0 to 600.3
Negative Likelihood Ratio	0.08	0.01 to 0.51
Positive Predictive Value	92.3%	62.9% to 98.8%
Negative Predictive Value	98.9%	93.3% to 99.8%
Accuracy	98.1%	93.3% to 99.8%
Cohen’s κ score	0.91	0.79 to 1.00
Females (*n* = 41)		
Sensitivity	100%	15.8% to 100%
Specificity	97.4%	85.5% to 99.9%
Positive Likelihood Ratio	39.0	5.6 to 270.0
Negative Likelihood Ratio	0	-
Positive Predictive Value	66.7%	22.4% to 93.3%
Negative Predictive Value	100%	-
Accuracy	97.6%	87.1% to 99.9%
Cohen’s κ score	0.79	0.39 to 1.00
Males (*n* = 64)		
Sensitivity	90.9%	58.7% to 100%
Specificity	100%	93.3% to 100%
Positive Likelihood Ratio	-	-
Negative Likelihood Ratio	0.09	0.01 to 0.59
Positive Predictive Value	100%	62.9% to 98.8%
Negative Predictive Value	98.1%	89.1% to 99.7%
Accuracy	98.4%	91.6% to 100%
Cohen’s κ score	0.94	0.83 to 1.00
≥65 years (*n* = 42)		
Sensitivity	100%	47.8% to 100%
Specificity	100%	90.5% to 100%
Positive Likelihood Ratio	-	-
Negative Likelihood Ratio	0	-
Positive Predictive Value	100%	-
Negative Predictive Value	100%	-
Accuracy	100%	91.6% to 100%
Cohen’s κ score	1.00	-
<65 years (*n* = 63)		
Sensitivity	87.5%	47.3% to 99.7%
Specificity	98.2%	90.3% to 99.9%
Positive Likelihood Ratio	48.1	6.8 to 341.5
Negative Likelihood Ratio	0.13	0.02 to 0.81
Positive Predictive Value	87.5%	49.7% to 98.0%
Negative Predictive Value	98.2%	89.6% to 99.7%
Accuracy	96.8%	89.6% to 99.7%
Cohen’s κ score	0.86	0.67 to 1.00

## Data Availability

The data supporting the findings of this study are available from the corresponding authors upon reasonable request.
